# Molecular Dynamics Simulations of the Breathing Phase Transition of MOF Nanocrystallites II: Explicitly Modeling the Pressure Medium

**DOI:** 10.3389/fchem.2021.757680

**Published:** 2021-10-25

**Authors:** Larissa Schaper, Julian Keupp, Rochus Schmid

**Affiliations:** Computational Materials Chemistry Group, Faculty of Chemistry and Biochemistry, Ruhr-Universität Bochum, Bochum, Germany

**Keywords:** MOF, nanocrystallites, molecular dynamics, barostat, force field, phase transition

## Abstract

One of the most investigated properties of porous crystalline metal-organic frameworks (MOFs) is their potential flexibility to undergo large changes in unit cell size upon guest adsorption or other stimuli, referred to as “breathing”. Computationally, such phase transitions are usually investigated using periodic boundary conditions, where the system’s volume can be controlled directly. However, we have recently shown that important aspects like the formation of a moving interface between the open and the closed pore form or the free energy barrier of the first-order phase transition and its size effects can best be investigated using non-periodic nanocrystallite (NC) models [Keupp et al. (Adv. Theory Simul., 2019, 2, 1900117)]. In this case, the application of pressure is not straightforward, and a distance constraint was used to mimic a mechanical strain enforcing the reaction coordinate. In contrast to this prior work, a mediating particle bath is used here to exert an isotropic hydrostatic pressure on the MOF nanocrystallites. The approach is inspired by the mercury nanoporosimetry used to compress flexible MOF powders. For such a mediating medium, parameters are presented that require a reasonable additional numerical effort and avoid unwanted diffusion of bath particles into the MOF pores. As a proof-of-concept, NCs of pillared-layer MOFs with different linkers and sizes are studied concerning their response to external pressure exerted by the bath. By this approach, an isotropic pressure on the NC can be applied in analogy to corresponding periodic simulations, without any bias for a specific mechanism. This allows a more realistic investigation of the breathing phase transformation of a MOF NC and further bridges the gap between experiment and simulation.

## 1 Introduction

One of the most intriguing features of the novel class of metal-organic frameworks (MOFs), also known as porous coordination polymers, is their structural flexibility combined with crystalline ordering. Kitagawa et al. coined the term 3rd generation MOFs for such systems, where the cell volume can change drastically upon external stimuli like pressure, guest adsorption, or other triggers (Kitagawa et al., 2004; Coudert, 2015). Especially the guest-induced breathing phase transition has initiated a lot of research since it substantially increases the working capacity when such porous materials are used in gas separation applications (Mason et al., 2015). One of the most intensively investigated MOF systems in this area is MIL-53, and we refer here to several reviews (Férey and Serre, 2009; Alhamami et al., 2014; Millange and Walton, 2018; Tomar and Singh, 2021). In this context, we want to note that it was Llewellyn et al. who have used mercury porosimetry, usually employed to characterize mesoporous systems, to trigger the open to closed pore phase transformation by a hydrostatic external pressure Beurroies et al. (2010). From a theoretical point of view, this phenomenon has been investigated in the context of small unit cell simulations with periodic boundary conditions (PBCs). However, there is meanwhile a good amount of evidence that the breathing effect depends on the size and also on the morphology of crystallites (Ehrling et al., 2021). In particular, it seems that only larger crystallites undergo this transformation, whereas smaller crystals behave rigidly. This tendency was even observed for surface mounted SURMOF thin films (Wannapaiboon et al., 2019). In order to investigate such effects by atomistic simulations, we recently started to perform extensive molecular dynamics simulations of MOF nanocrystallites (NCs) using the first principle parameterized force field MOF-FF (Bureekaew et al., 2013; Dürholt et al., 2019). Compared with conventional PBC simulations, it became evident that the phase transition is not occurring in a concerted fashion over the whole system but often starts in the outer pores and travels through the system. As a consequence, both the closed and the open pore phase are present in the system. In recent force field simulations using large super cells in PBC, Rogge et al. (2019) made similar observations. For NCs of increasing size, a size effect could already be observed for the transformation barrier, indicating a surface resistance for the overall process, which would indeed explain the experimentally observed trends. However, it needs to be kept in mind that the simulated NCs are still substantially smaller than the experimentally investigated systems and the chosen surface termination of the NCs must be considered idealistic (Zacher et al., 2011; Amirjalayer et al., 2014). In PBC, the volume is well defined by the simulation cell volume, and the pressure can be computed from the virial. A barostat can be invoked and advanced techniques allow to sample the internal pressure of a MOF with changing cell shape but fixed volume (Rogge et al., 2015). In contrast, for the irregular-shaped NCs, the volume is not well defined. It is possible to open a closed pore NC system by increasing the temperature in a physically meaningful way. However, in the previous work, a constraining mechanical force, acting on a selected set of atoms (SBUs on opposite edges of the NC), was used to imitate the effect of pressure on the NCs (Keupp and Schmid, 2019). This allows to define a reaction coordinate for the phase transformation and to compute free energy profiles using umbrella sampling. In contrast to the thermal opening, these artificial forces have nothing in common with a hydrostatic pressure acting equally on the surface of the NC and the resulting mechanism could be biased by the choice of the RC. Thus, it would be desirable to be able to apply an isotropic hydrostatic pressure on the NC without any such bias. To achieve this, a procedure inspired by the experimentally well-established mercury porosimetry was developed. The non-wetting and high surface tension liquid mercury is typically used to determine the pore size distribution of mesoporous materials. Llewelyn et al., however, applied it to measure the breathing transformation of the nanoporous MOFs MIL-53, where the medium is not able to enter the pores, but the hydrostatic pressure triggers the pore closing (Rodriguez et al., 2016). For an atomistic simulation of an NC, embedded in such a fluid, an additional numerical effort is needed to compute the interactions of the additional pressure bath particles. In addition, the medium should be “non-wetting” in the sense that a high particle-particle interaction and a mostly repulsive MOF-particle interaction prevent any loss of pressure bath particles into the empty MOF pore space, which could also bias the transformation mechanism.

**FIGURE 1:**
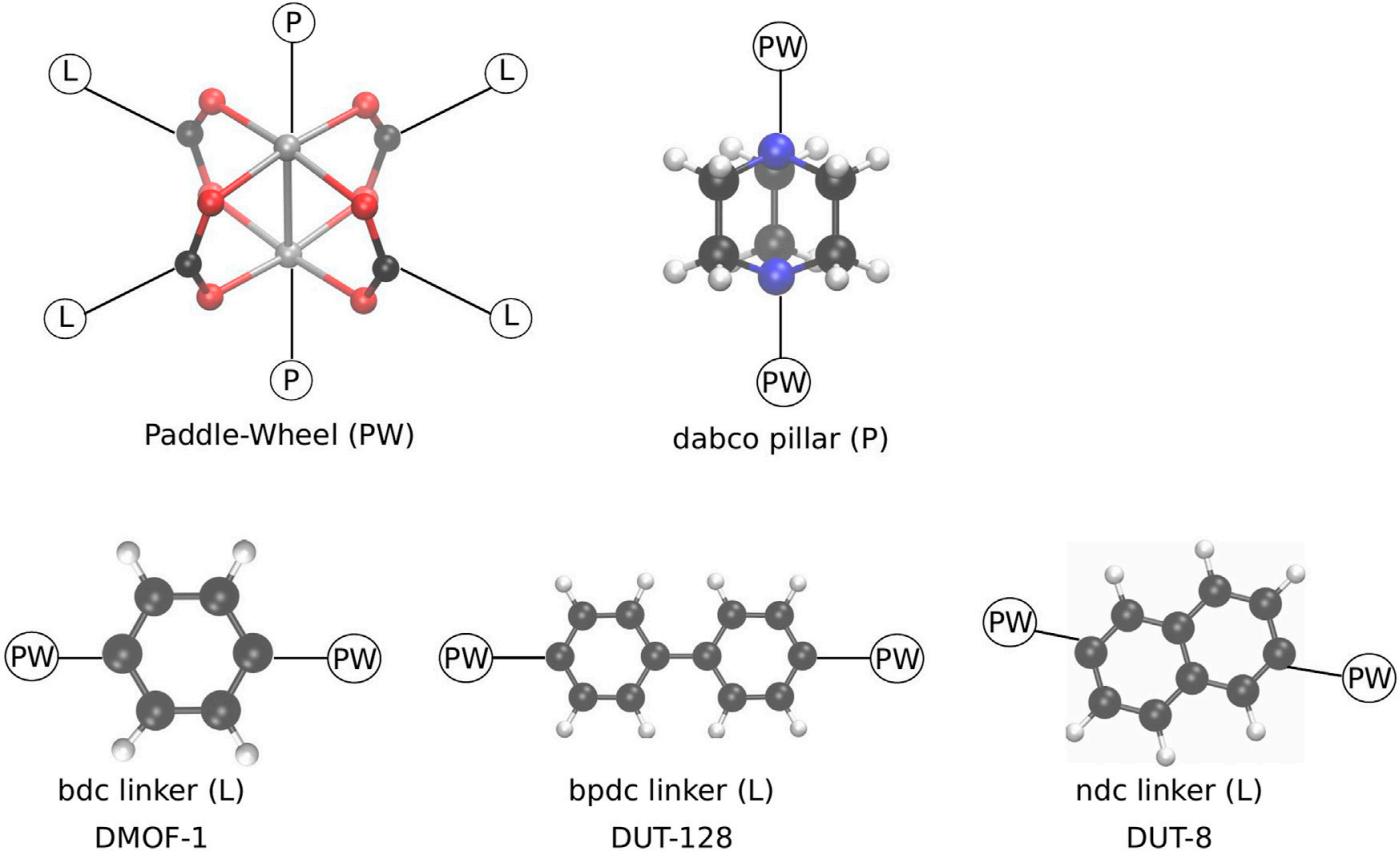
Schematic representation of the building blocks, forming the pillared layer MOFs investigated in this study. In the paddle-wheel unit (PW) the metal atoms (grey) are either Zn or Cu. The pillar (P) is always fixed, whereas depending on the chosen linker (L) a different MOF is formed in the same basic pcu topology.

In this contribution, a proof-of-concept study of such a mediating pressure bath is presented. It is shown that such a mediating medium can be used to close MOF NCs, and that the previously observed closing mechanism is indeed not due to the chosen RC. For this purpose, the same model system, DMOF-1 [Zn_2_(bdc)_2_(dabco); bdc: 1,4-benzenedicarboxylate; dabco: 1,4-diazabicyclo(2.2.2)octane] (Dybtsev et al., 2004) is chosen. In addition, NCs of the MOFs DUT-8(Cu) [Cu_2_(2,6-ndc)_2_(dabco); DUT: Dresden University of Technology; 2,6-ndc: 2,6-naphthalenedicarboxylate] (Klein et al., 2010), and DUT-128(Zn) [Zn_2_(4,4′-bpdc)_2_(dabco); 4,4′-bpdc: 4,4′-biphenyldicarboxylate] (Bönisch et al., 2021) are investigated in order to reveal the effect of a different linker group on the process of pore closing (see Figure 1).

**FIGURE 2:**
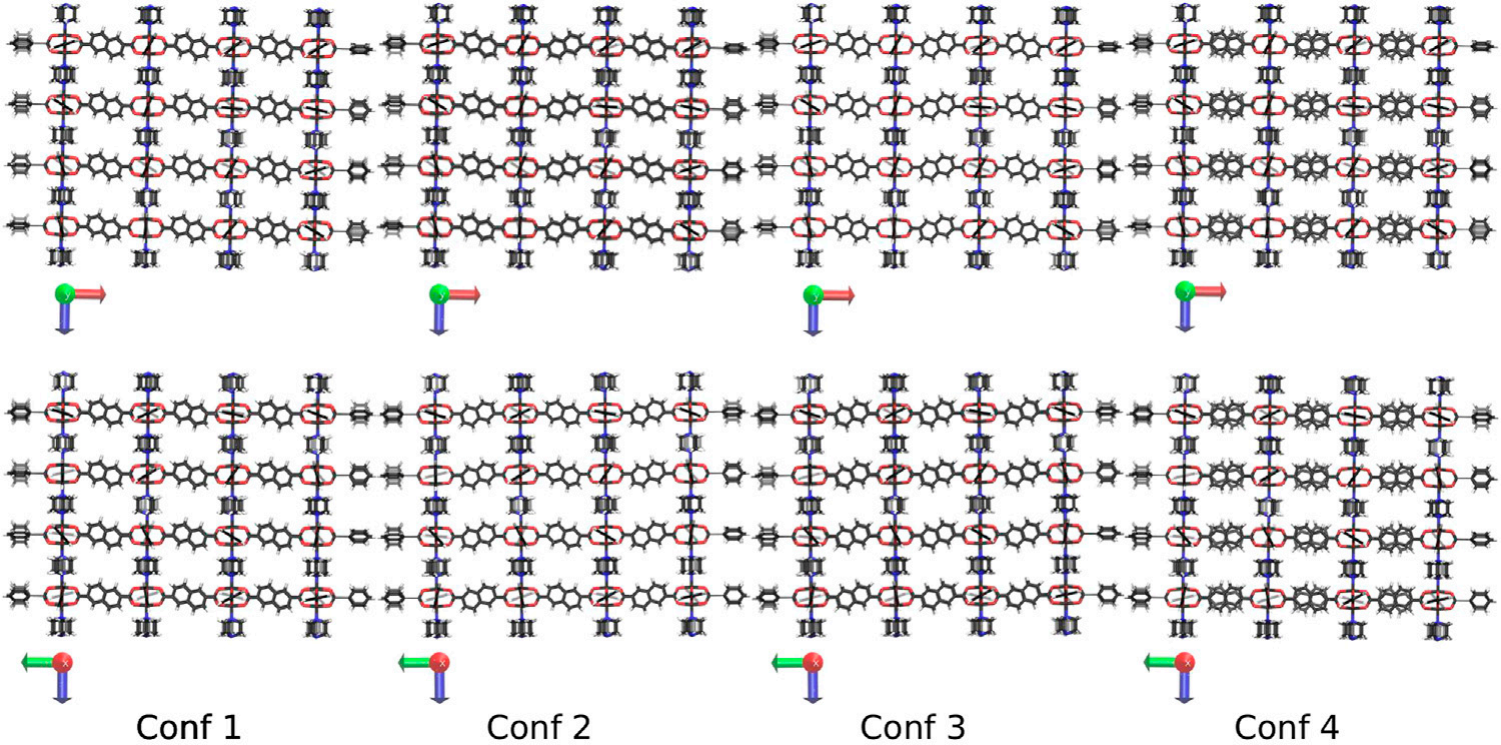
The four investigated conformations of DUT-8 shown along the y-axis **(up)** and the x-axis **(down).** The scheme how to orient the ndc linker is kept constant if the system size is increased. In the fourth conformation, the linker in each second row is flipped by 180°.

Due to the reduced symmetry of the ndc linker, different conformations how to build the DUT-8 NCs are possible. In this study, only four conformations are considered, which are kept fixed for increasing system sizes. The four different conformations are shown in Figure 2 for the 4 × 4 × 4 NC as an example. All four conformations fulfill the zero net sum criterion proposed by Goodwin et al. (Reynolds et al., 2021).

## 2 Computational Details

Differently sized nanocrystallites (NCs) of DMOF-1, DUT-8(Cu), and DUT-128(Zn) were used as initial structures to prepare the simulation setup, containing one NC and the pressure bath atoms. The NCs were constructed in the same way as it is described in reference (Keupp and Schmid, 2019). They are named by the number of paddle-wheel units in each spatial direction, i.e., 4 × 4 × 4 means that the NC has four zinc paddle-wheels and three orthorhombic pores respectively in the x-,y-, and z-direction. For this proof-of-concept study, more or less cubic NCs with an equal number of PW units in all spatial directions were used, but simulations on non-isotropic NCs are underway. The open pore form of the NCs was centered in a cubic box that is large enough for the NCs, including the phenyl and pillar surface terminating groups and additional buffer distance of 12 Å. The box was filled with pressure bath atoms under the condition not to place them inside the NC’s pore using the packmol program package (Martínez et al., 2009). All force field calculations were performed with the LAMMPS program package (Plimpton, 1995), (https://lammps.sandia.gov). The interactions of the DMOF-1, DUT-8, and DUT-128 were described by the first principles parameterized MOF-FF force field (Bureekaew et al., 2013; Dürholt et al., 2019). The DMOF-1 systems were structurally optimized and equilibrated for 50 ps with a time step of 1 fs in the NVT ensemble at T = 300 K. The system was coupled to the Nosé-Hoover thermostat (Hoover, 1985) with a relaxation time of 0.05 ps and a temperature of 300 K, whereby the temperature is only a reference for the kinetic energy in the system. The initial velocity was generated by a Maxwell Boltzmann distribution. The DUT-8 and DUT-128 NCs were heated up from 10 to 300 K at a pressure of 0 bar during equilibration for 50 ps with a time step of 1 fs in the NpT ensemble. The thermostat relaxation time was set to 0.05 ps and the barostat relaxation time to 1 ps. In this study, all NpT simulations were carried outusing the Nosé-Hoover barostat implemented in LAMMPS (Parrinello and Rahman, 1981; Martyna et al., 1994; Shinoda et al., 2004; Tuckerman et al., 2006). The procedure described in the following was carried out for all investigated systems. A subsequent NpT run was performed at a pressure of 0 bar and a temperature of 300 K for 1 ns with a time step of 1 fs. The thermostat relaxation time was set to 0.1 ps and the barostat relaxation time to 1 ps. The pressure ramp simulations were carried out under the same conditions as the previous NpT run for 3 ns while the pressure is linearly increased from 0 to 3,000 bar. The trajectory contains structures of each 0.5 ps time step. For the volume analysis, the volumes of the individual pores were calculated using the qhull library within scipy (Barber et al., 1996). First, the center of mass of each paddle-wheel is determined for each of the chosen frames of the trajectory of the pressure ramp simulation, respectively. The volume of an individual pore is obtained by calculating the volume of the convex hull spanned by eight paddle-wheels that define a pore in the NC. Detailed information can be found in the Supplementary Material.

## 3 Results and Discussion

### 3.1 Pressure Bath Medium

In the presented procedure, a so-called pressure bath is introduced, which mediates an isotropic hydrostatic pressure to the considered NC. The simulation setup requires some preliminary considerations concerning the pressure bath medium. For the force field calculation, suitable van-der-Waals parameters, the minimum distance r_0_ and the well-depth *ϵ* of the pressure bath atoms are needed. Since the pressure bath is a hypothetical medium, which only represents a fluid pressing on the MOF, the modeled fluid does not necessarily comply with the thermodynamic properties of natural elements, i.e., mercury. Note, that the properties of mercury cannot accurately be modeled with a simple two-body potential like a Lennard-Jones term (Iakovlev et al., 2015). Therefore, in this study, the focus was on choosing the parameters such that the structural transition from open to closed pores using a pressure bath is simulated efficiently. For this purpose, a screening experiment for different minimum distance r_0_ and well-depth *ϵ* parameter combinations was performed using the 4 × 4 × 4 NC as a test system. For every tested value, an NpT MD simulation of 1 ns with zero pressure was carried out, in which the stability of the MOF in the pressure medium and the simulation speed were analyzed. Note, that the screened parameters were not optimized but only selected to fulfill the following criteria: 1) The parameters should be suitable to adequately trigger the pore closing starting from a completely open pore form, 2) they should not require great numerical effort, and 3) they should avoid the diffusion of pressure bath particles into the MOF pores. It was started using parameters inspired by known Lennard-Jones parameters of mercury (Bomont and Bretonnet, 2006), but with an about doubled particle radius. The screening experiment resulted in r = 3.0 Åand *ϵ* = 0.8 kcal/mol as a solid option fulfilling the requirements. Other parameter combinations, e.g., choosing larger *ϵ*-values up to *ϵ* = 1.7 kcal/mol, rendered the immersed open-pore phase of the MOF unstable, i.e., the NC pores are partially closed without any external pressure increasing. Furthermore, such a large particle can fill the void around the NC relatively efficient which results in an adequate numerical performance of 7.652 ns/day (32 processors; 10190 atoms total, thereof 4160 MOF atoms and 6,030 pressure bath atoms) compared to smaller *ϵ*-values.

Since this approach is inspired by mercury porosimetry, the pressure bath atoms were characterized as hypothetical mercury atoms, i.e., they have an atomic mass equal to that of mercury. However, the particles are substantially larger (about twice the minimum distance) than in other force fields for mercury. An important aspect for the setup of the simulation is to determine the proper number of pressure bath atoms in the simulation box and to avoid any dense packing. To ensure this, the number of pressure bath atoms is determined from the low pressure particle density and the remaining void space around the MOF. In order to compute the particle density of the pure pressure bath medium, an NpT simulation at T = 300 K and a zero pressure was performed for 5 ns in a cubic box. Averaging gives a mean particle density of 5.65⋅10^−3^ Å^−3^. This low pressure mean particle density was used to setup all simulations, i.e., the free volume in the simulation box not covered by the MOF NC was filled with the respective number of pressure bath particles.

**FIGURE 3:**
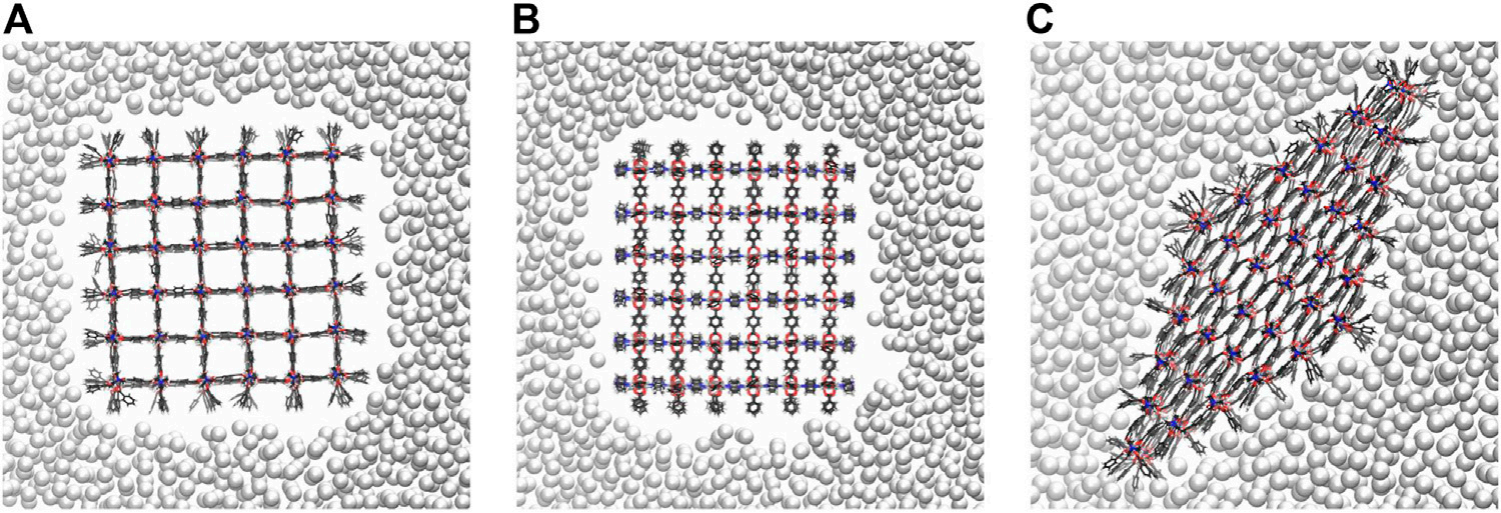
The nearly cubic 6 × 6 × 6 NC in the pressure bath medium viewed along the z-axis **(A)** and the x-axis **(B)**. If no external pressure is applied, the pressure bath atoms do not move inside the MOF pores, forming a cavity around the NC. In the closed pore form **(C)**, the pressure bath atoms approach closer to the NC, interacting more strong with the surface groups. To visualize this, pressure bath atoms in front or behind the NC are not shown using clipping planes.

**FIGURE 4:**
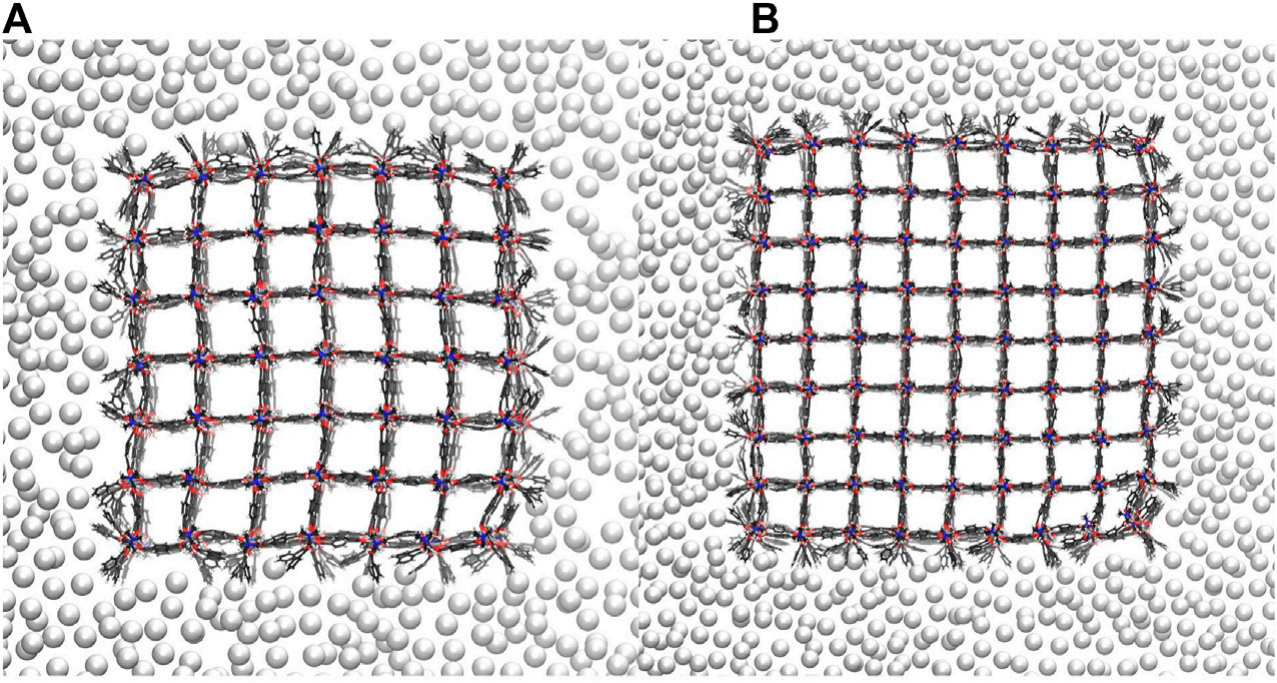
The pressure bath atoms mediate an external pressure of 980 bar to the 7 × 7 × 7 NC **(A)** and the 9 × 9 × 9 NC **(B)**. Both NCs are shown along the z-axis. No pressure bath atoms enter the MOF pores. Instead, the pressure bath medium keeps the cavity around the NCs so that the pressure is strongest at the corners of the NCs, which leads to a local deformation.

In experiments, mercury does not penetrate the pores of microporous materials. Thus, it is required that the pressure bath atoms never move inside the pores of the NC during optimization and MD simulation. To ensure this, the attractive intermolecular interactions between the pressure bath medium and the MOF atoms are disabled, i.e., there is only a repulsive interaction. Figure 3 shows a snapshot of the 6 × 6 × 6 NC in the pressure bath medium along the z-axis (see Figure 3A) and the x-axis (see Figure 3B) when no external pressure is applied. It is obvious that there are no pressure bath atoms inside the MOF pores, but the medium closely surrounds the NC. The interactions in the pressure bath medium and the resulting surface tension are sufficiently strong to keep the pressure bath atoms to remain in the bulk. Also, upon an increasing the external pressure, the bath atoms do not enter the pores but stick even closer around the NC. This leads to the strongest pressure at the corners of the NC, which results in a deformation of these pores. The effect is more significant for larger-sized NCs. For example, in Figure 4, it is shown that the pores at the corners of the 7 × 7 × 7 NC (see 4a) and the 9 × 9 × 9 NC (see 4b) have a nearly closed pore form. Therefore, the pressure bath medium fulfills the requirements of a non-wetting fluid, i.e., it does not enter the MOF pores but only mediates external pressures. It is a characteristic property of the pressure bath medium to keep the pressure bath atoms in bulk and form a cavity around the NC during pressure increase.

### 3.2 Proof of Concept: The Model System DMOF-1

**FIGURE 5:**
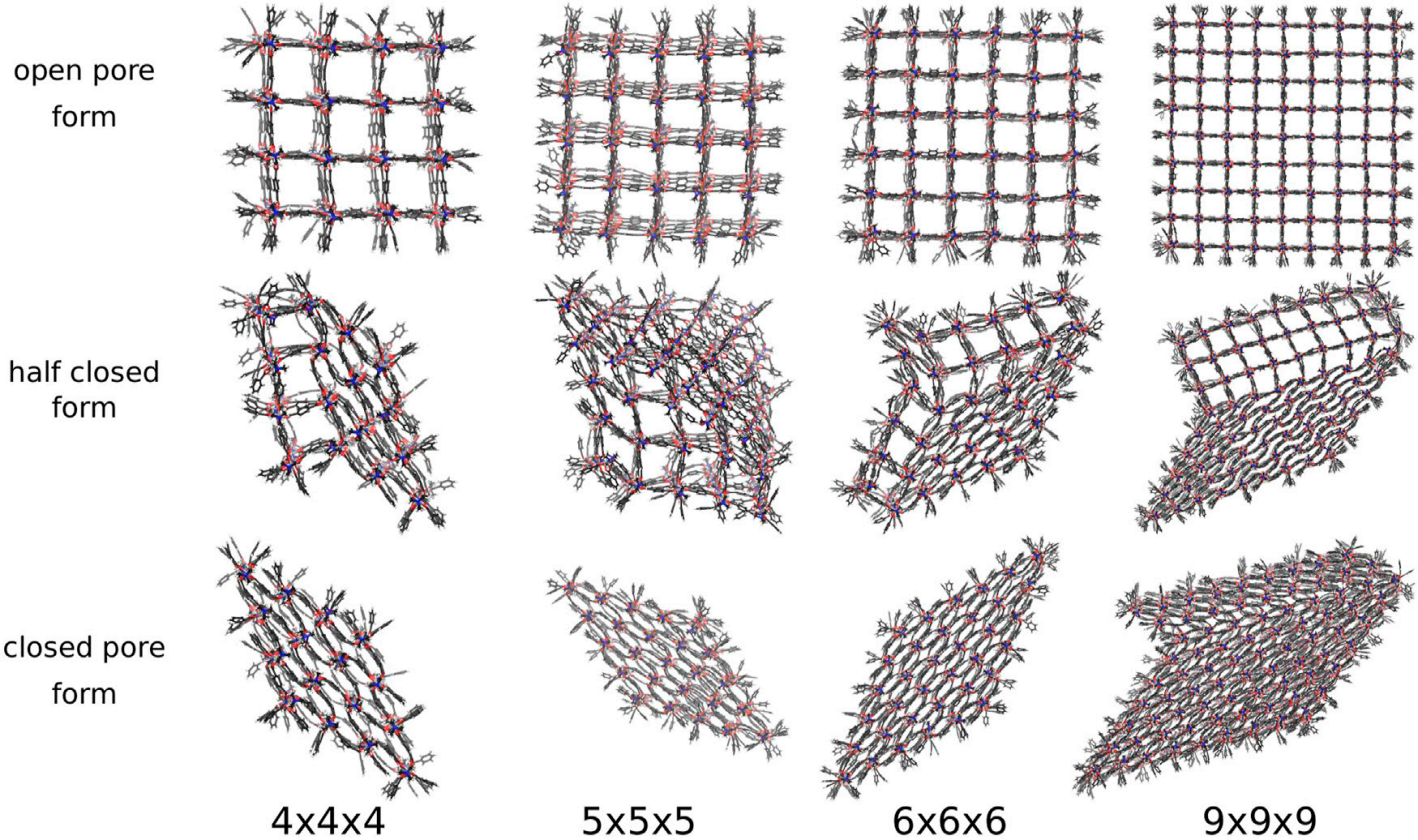
The open, half closed and closed pore form of differently sized DMOF-1 NCs. The snapshots are taken from the respective pressure ramp simulation of the structural transition from the open to the closed pore form induced by the pressure bath medium. Two differently shaped closed pore forms and four differently shaped transition structures are observed.

The new pressure bath method is now applied to seven differently sized DMOF-1 NCs, i.e., a pressure ramp simulation is performed once with the 3 × 3 × 3 NC up to the 9 × 9 × 9 NC. In the previous study, where a distance constraint is used to induce the phase transition, always a perfectly diamond-shaped closed pore form is received (Keupp and Schmid, 2019), which is expected to be the favored closed pore structure. Figure 5 shows the open, half closed and closed pore forms of the 4 × 4 × 4, the 5 × 5 × 5, the 6 × 6 × 6, and the 9 × 9 × 9 NCs. These structures represent the different pathways observed for all investigated sizes. The snapshots are taken from simulations in which an external pressure is linearly increased so that an increasing isotropic hydrostatic pressure is applied to the open pore form of the respective NC until a structural transition to the closed pore form occurs. Since the pore closing propagates for all investigated systems through the xy-plane for all z-layers, it is sufficient to simplify the process by looking along the z-direction. Two differently shaped closed pore forms can be observed. One closed pore form is diamond-shaped, resulting from the collective pore closing along the same spatial diagonal. In the other case not all pores close along the same spatial diagonal (see Figure 5, closed pore form of the 9 × 9 × 9 NC), which causes the diamond-shaped structure to be disturbed. For the smaller NCs up to the 6 × 6 × 6 NC, the diamond-shaped closed pore form is observed, whereas the larger NCs up to the 9 × 9 × 9 NC end up in the disturbed diamond-shaped structure. This final product is likely to be kinetically trapped, but such domains with different folding directions are more likely to be formed in larger crystallites as investigated experimentally. The observation of a structural transition confirms that the approach of using a pressure bath medium to mediate an external pressure to MOF NCs is a valid concept to initialize the breathing phase transition and imposes no artificial bias on the closing mechanism.

A detailed analysis of the simulations with the pressure bath revealed also two major disadvantages of the new method. First, due to high fluctuations in the pressure bath medium the integration of pressure-volume curves (Rogge et al., 2015) unfortunately yields inconclusive free energy profiles. Additionally, since the attractive interactions had to be disabled to avoid permeation of the pressure bath atom into the pores, effective negative pressures do not significantly impact the pore behavior. Thus, pore opening can be triggered only by increasing temperature (as in experiment) but not by negative pressure.

**FIGURE 6:**
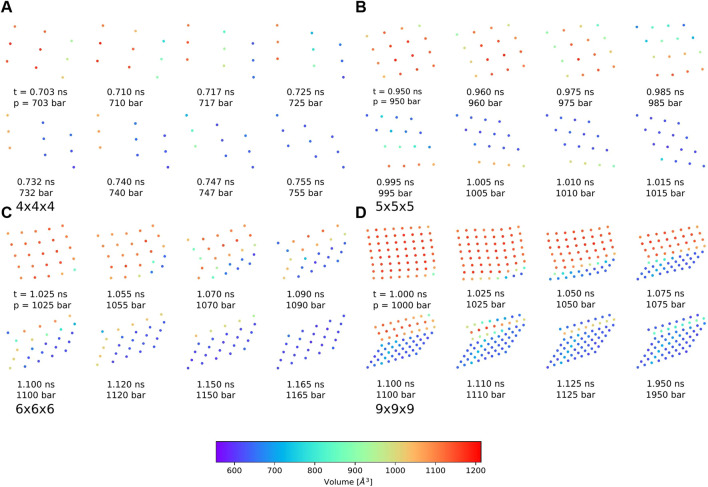
Pore size analysis of the differently sized DMOF-1 nanocrystallites for different snapshots of the phase transition from open to closed pore structure averaged along the z-axis and viewed on the xy-plane. The centers of the pores are colored according to the respective size. **(A)** For the 4 × 4 × 4 NC, the phase transition starts in one corner and travels first in one direction. Then, a layer-by-layer closing along the other direction is observed. The closed pore form is the expected diamond-shaped structure. **(B)** For the 5 × 5 × 5 NC, the phase transition occurs from two corners and first travels to the central layers. All pores close along the same spatial diagonal so that the closed pore form shows the expected diamond-shaped structure. **(C)** For the 6 × 6 × 6 NC, the pore closing starts in one corner and propagates in both directions until a pore closing along a different spatial direction occurs in the upper left corner. A reopening of these pores leads to the expected diamond-shaped closed pore structure. **(D)** For the 9 × 9 × 9 NC, the pore closing mechanism is similar to those of the 6 × 6 × 6 NC, but no reopening is observed. The closed pore form nearly shows the expected diamond-shaped structure. Two layers disturb the symmetry because these pores are closed along a different spatial diagonal. This leads to a row of pores with a slightly larger pore volume of approximately 850 Å^3^.

Nevertheless, the pressure bath method facilitates the investigation of the phase transitions of MOF NCs without any bias on the mechanism and allows to determine the necessary external pressure to initialize the pore closing. In addition, the atomistic mechanism can be studied like for example which pores closes first, and how the interface propagates through the system. In this work, four possible phase transition mechanisms could be observed in which the pore closing proceeds layer-by-layer. One in which the pore closing starts from one corner (shown in Figure 5 for the 4 × 4 × 4 NC), or where the pore closing starts from two corners either along the same spatial diagonal (shown in Figure 5 for the 5 × 5 × 5 NC) or along two different spatial diagonals (shown in Figure 5 for the 6 × 6 × 6 NC and the 9 × 9 × 9 NC). Although the 6 × 6 × 6 NC and the 9 × 9 × 9 NC start their pore closing mechanism similarly, i.e., the pore closing occurs from two opposite corners, they end up in different closed pore structures. This is caused by a reopening of pores in the 6 × 6 × 6 NC, whereas the pores of the 9 × 9 × 9 NC stay closed. In order to elucidate the phase transition mechanism, the individual pore volume of the 4 × 4 × 4, the 5 × 5 × 5, the 6 × 6 × 6, and the 9 × 9 × 9 NC was calculated for significant frames. The pore size is averaged along the z-axis so that the view on the xy-plane is shown. The center of the pores is colored according to the respective pore volume. Blue-colored pores represent closed pores with a volume of approximately 610 Å^3^, whereas red-colored pores represent the open pores with a volume of approximately 1170 Å^3^. Figure 6A shows the pore size analysis of the structural transition of the 4 × 4 × 4 system to point out the first observed pore closing mechanism. At t = 0.703 ns, it can be seen that the transition from open to the closed pore form starts at the edge of one side of the NC, i.e., one outer row of three pores closes first. This initializes the closing of the middle layer at t = 0.717 ns up to t = 0.732 ns. On the opposite side of NC, the pores close last, as shown for t = 0.740 ns up to t = 0.755 ns. Therefore, the structural transition occurs from one outer side through the 4 × 4 × 4 NC to the opposite side. The pores do not close synchronously but successively from side to side of the crystal. This agrees with the previously performed closing by mechanical forces (Keupp and Schmid, 2019), which revealed a similar mechanism for the phase transition. However, using the pressure bath medium to initialize the phase transition can also result in another pathway, as it is shown in Figure 6B for the 5 × 5 × 5 NC. For t = 0.960 ns, the pores at the two upper corners close simultaneously. This observation differs from the mechanism obtained by mechanical forces (Keupp and Schmid, 2019). At t = 0.985 ns, the pore closing travels to middle-layer pores, i.e., the pore closing goes from outer to inner pores. From t = 0.995 ns up to t = 1.015 ns, a layer-by-layer closing is observed so that the closed pore form ends up in the expected diamond-shaped structure. A third possible mechanism for the phase transition is observed for the 6 × 6 × 6 NC. This pathway includes characteristics of both of the formerly described scenarios. Figure 6C clarifies that the closing occurs from one corner of the 6 × 6 × 6 NC, which is similar to the first described pathway, i.e., the propagation proceeds in one direction up to t = 1.070 ns. At this point, the pores on the left side close. This pore closing is pressure mediated and not induced by adjacent closed pores. At t = 1.090 ns, the pores in the upper left corner are completely closed. This can be explained by the usage of the pressure bath and the linear pressure ramp. During the simulation, the pressure is applied equally to the system from all directions. This stimulates the flexible pores at the edges of the NC first to avoid the increasing pressure. Especially for larger systems, it seems to be structurally more favorable to close the pores at different sides to avoid the pressure in the system instead of waiting for the impulse that causes the closing of the pores to be propagated from one side of the NC to the opposite one. Since the pores in the upper left close along a different spatial diagonal than the remaining closed pores, the left side of the NC bends into the structure. From t = 1.090 ns up to t = 1.120 ns, two phase boundaries are observed, i.e., closed pores are adjacent to open pores which are next to closed pores. From t = 1.110 ns up to t = 1.150 ns, the pores in the upper left corner reopen again, and its pore volume increases from nearly 700 Å^3^ up to 1050 Å^3^. This can be caused by the fact that this pore first closes along a different spatial diagonal than the remaining pores. With increasing pressure, it seems favorable to close all pores along the same spatial diagonal to end up in a nearly perfect diamond-shaped structure at t = 1.165 ns. The pore size analysis of the 9 × 9 × 9 NC, shown in Figure 6D, results in an approximately equal mechanism for the phase transition than the 6 × 6 × 6 NC. It starts in the lower right corner, propagates along the edge in one direction at t = 1.000 ns up to t = 1.050 ns and, at t = 1.075 ns also in the other direction. From t = 1.075 ns up to the end of the simulation, the NC bends on the left side into the structure due to the border between already closed and still open pores of the NC. At t = 1.100 ns, a simultaneous pore closing is observed, i.e., the middle layers and the pore in the upper right corner close. The upper right pore is closed along a different spatial diagonal. Up to t = 1.125 ns, the NC pores close simultaneously from both sides of the NC, where the top layer continues closing along a different spatial diagonal. In contrast to the third described pathway, the pores closing along a different spatial diagonal than the majority of the layers do not reopen again. This leads to a disturbed diamond-shaped closed pore form in which the pores of one layer have a larger pore volume of 850 Å^3^ than the remaining pores. Higher pressures are needed to fold them further, but the bending on the left side persists.

Therefore, for the “breathing” phase transition mechanism of the model system, different pathways and different closed pore forms can be observed if the pressure bath method is applied. This is in contrast to the investigation of the phase transition by mechanical forces, in which only one mechanism and one closed pore form is presented (Keupp and Schmid, 2019). The pressure bath method does not bias the pore closing mechanism and enables to explore the potential energy surface of the phase transition more flexibly, i.e., it takes fluctuating configurations of the closed pore forms into account. To find the optimum closed pore form, slower pressure progression leaves the system a greater chance to react to the external force resulting in phase transitions that are closer to the true minimum reaction energy path. Since the calculations were only performed once for the most systems, it cannot be validated if the pathway of the pore closing and the resulting closed pore form depends on the size of the NC. For this purpose, more statistics would have to be performed. This proof-of-concept study only demonstrates that differently shaped closed pore forms are observable if the pressure bath method is used in contrast to the previous artificial distance restraint.

To further verify this, a series of five pressure ramp simulations for the 6 × 6 × 6 DMOF-1 NC reveals that different pore closing mechanisms ending up in similar or different closed pore forms can occur for the same system size with different initialization, i.e., with different positions of the pressure bath atoms in the initial system. In addition to the formerly described pore closing mechanism for the 6 × 6 × 6 NC, the layer-by-layer pore closing mechanism ending up in the diamond-shaped closed pore form as described for the 4 × 4 × 4 NC, as well as the pore closing mechanism described for the 9 × 9 × 9 NC ending up in the disturbed diamond-shaped structure, is observed for the differently initialized 6 × 6 × 6 NCs as well. However, all pressure ramp simulations of the 6 × 6 × 6 NC have in common that the mediated pressure initiating the pore closing is in the same range between 1 and 1.1 kbar, indicating that the limiting pressure for the pore closing is independent of the mechanism. Despite this, the size of the NC affects the external pressure needed to initialize the pore closing. Table 1 shows the pressure needed to initialize the transition to the closed pore form. The smaller systems start to close their pores at lower pressures than larger systems, which close at approximately the same pressure of 1 kbar. This rapid convergence of the limiting pressure around 6 × 6 × 6 is somewhat surprising. Note that for DMOF-1 experimentally, no stable closed pore form is observed, but for the corresponding (slightly more stiff) Cu-DMOF-1, a pressure of about 2 kbar is needed for pressure-induced amorphization (Vervoorts et al., 2021). Simulations with MOF-FF in periodic boundary conditions gave the same limiting pressure of 2 kbar from the computed pV equation of state (EOS), but in NpT pressure ramp simulations, phase transformations are observed just above 1 kbar (Vervoorts et al., 2021). The fact that fluctuations in NpT simulations lead to a pore closing at pressures below the limiting pressure from pV-EOS is well known (Rogge et al., 2015). Because of these reasons and the fact that the energy between the open and closed phase and the free energy barrier also converge already at about a size of 6 × 6 × 6 of the NCs (Keupp and Schmid, 2019), the limiting pressure seems indeed to be converged. As a working hypothesis, this is attributed to the fact that a certain amount of pores need to initiate the closing simultaneously and that this number is already reached for this NC size.

**TABLE 1:** The pressures for each system size of DMOF-1 NCs at which the phase transition starts. If the system size is increased from the smallest system to the 6 × 6 × 6 NC, higher pressures are needed to initialize the closing, whereas the larger systems need nearly the same pressure as the 6 × 6 × 6 system.

System	Pressure (bar)
3 × 3 × 3	645
4 × 4 × 4	703
5 × 5 × 5	950
6 × 6 × 6	1025
7 × 7 × 7	1100
8 × 8 × 8	1025
9 × 9 × 9	1000

### 3.3 Application to Breathing MOFs: DUT-128 and DUT-8

Since the pressure bath method works as expected for the model system DMOF-1, the study was extended to selected conformations of DUT-8(Cu) NCs, as well as to DUT-128(Zn) NCs, to investigate the effect of different linkers on the process of pore closing. In contrast to the model system, for these MOFs stable closed pore phases are observed experimentally, due to the stronger dispersive interactions of the larger linkers (see Figure 1). However, as already pointed out, the here simulated NCs with a size of up to around 10 nm are substantially smaller than what is typically studied experimentally. In a recent investigation, DUT-8(Ni) and DUT-8(Co) powders with a crystallite size of 50–200 nm were found to stay in the open pore form when loading with guests, whereas only larger systems in the range of 300 *μ*m appeared to be flexible (Ehrling et al., 2019). Thus, in non-ideal real systems with defects, impurities, and less ordered surface termination, size effects appear in substantially larger dimensions than the here simulated systems. The intention here is mainly to compare the different MOFs on an equal footing. As expected, the external pressure needed to initialize the phase transition of DUT-8 NCs is smaller than that for DMOF-1 NCs. However, the different DUT-8 conformations do not have a sizable impact on the phase transition onset (see Table 2).

**TABLE 2:** The applied external pressures for each system size of DUT-8 NCs at which the phase transition starts. In general, the external pressures are nearly in the same range from approximately 400–800 bar for all investigated systems, independent of the conformation. For an increasing system size the external pressure has to be slightly higher, but the variation is smaller compared to the model system.

System	Pressure (bar) Conf 1	Pressure (bar) Conf 2	Pressure (bar) Conf 3	Pressure (bar) Conf 4
3 × 3 × 3	550	485	398	508
4 × 4 × 4	615	625	635	568
5 × 5 × 5	698	748	720	685
6 × 6 × 6	700	770	775	675
7 × 7 × 7	750	745	750	745
8 × 8 × 8	810	815	785	785
9 × 9 × 9	780	790	855	875

**FIGURE 7:**
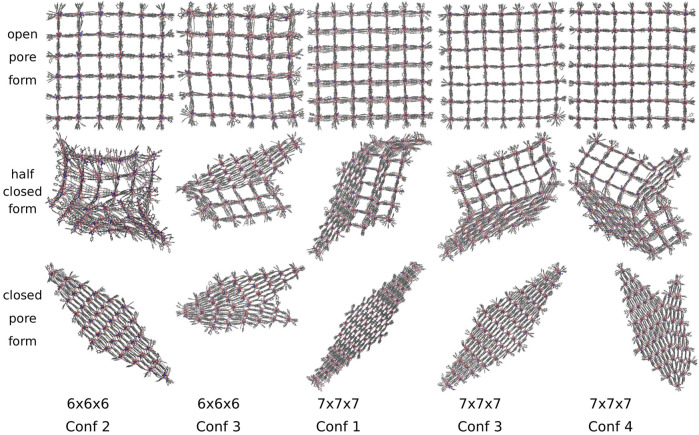
The open, half closed and closed pore form of differently sized DUT-8 NCs. Two differently shaped closed pore forms and five transition structures are observed. The closed pore forms are equivalent to those observed for the model system.

DUT-8 NCs adapt two differently shaped closed pore forms, converting along five different transition structures shown in Figure 7. Out of 28 simulations, 16 end up in a diamond-shaped and 12 in a disturbed diamond-shaped closed pore form, independent of the conformation and the system size. The observation of differently shaped closed pore forms agrees with already reported deviations from the perfect periodic image (Melix et al., 2019). The DUT-8 NCs show an equivalent number of different closed pore forms as the DMOF-1 model system. Three out of five transition structures and pore closing mechanisms are similar to those of the model system.

Additionally, larger DUT-8 NCs reveal additional transition pathways, as shown in Figure 7 for the 7 × 7 × 7 NC with conformation 1 and conformation 4. In the transition structure of conformation 4, the pores at two opposite corners are closed along different spatial diagonals. In contrast to the 6 × 6 × 6 NC of DMOF-1, the DUT-8 NC does not reopen but ends up in a disturbed diamond-shaped structure. This could be caused by the progress of the pore closing, i.e., for this number of pores already closed along another spatial diagonal, it seems not to be possible to reopen again. For this case, it might be interesting to investigate the effect of a slower pressure increase to give the system the time to reopen and to close along another spatial diagonal again. For further research, it has to be figured out more precisely under which conditions the systems can reopen their pores again. The last transition structure shown for the 7 × 7 × 7 NC with conformation 1 has no equivalent DMOF-1 transition structure since a middle layer, adjacent to the open pore layer, closes. This case is not observed for DMOF-1, but it was observed in fu-MOF NC simulations (Keupp et al., 2021).

**FIGURE 8:**
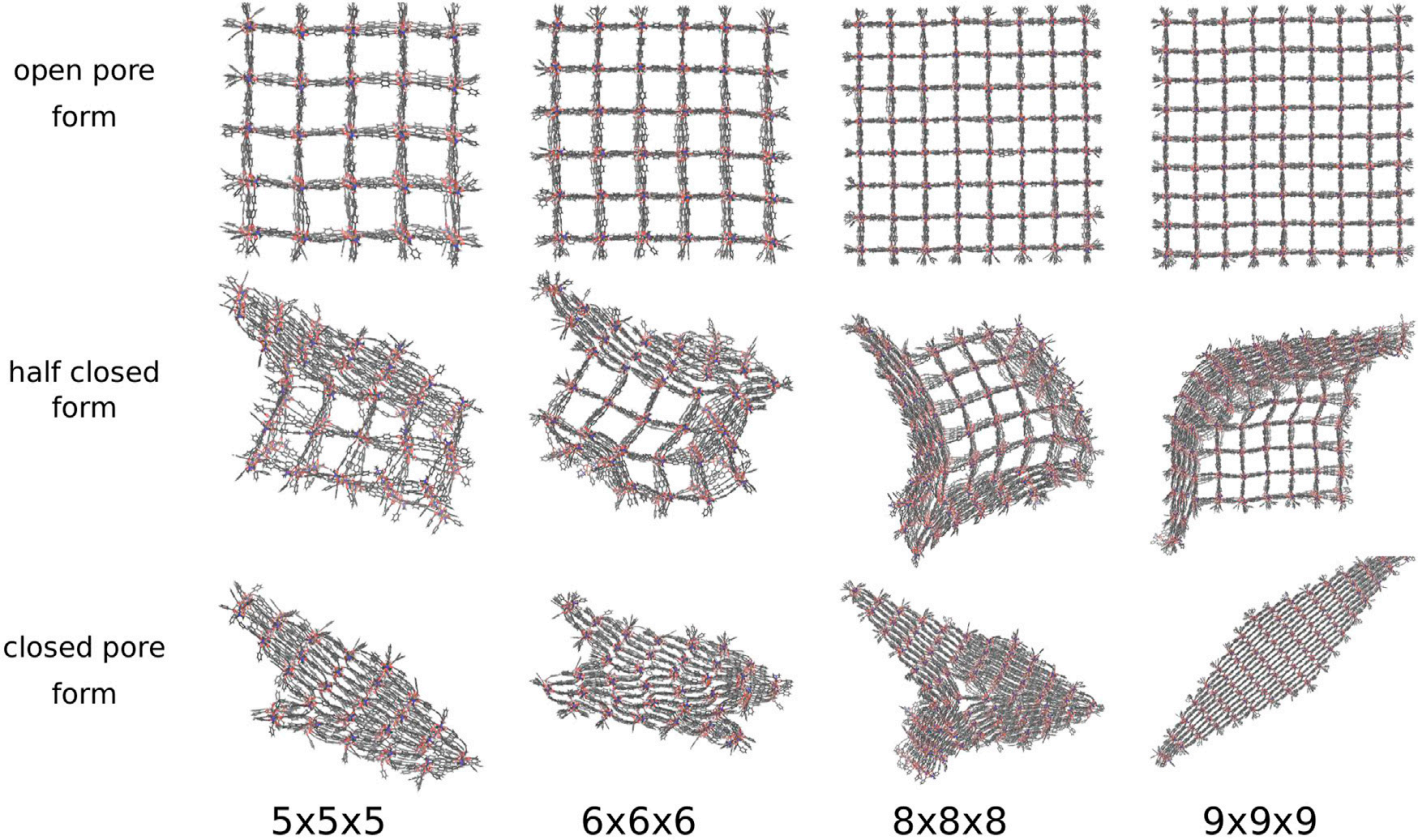
The open, half closed and closed pore form of differently sized DUT-128 NCs. Four differently shaped closed pore forms and transition structures are observed. In addition to the diamond-shaped structure and the disturbed diamond-shaped structure, a Z-shaped and a T-shaped structure is observed.

For DUT-128 (Zn), the NCs with the sizes 3 × 3 × 3 up to 9 × 9 × 9 were investigated in the same manner by adding the pressure bath medium and applying a linearly increasing external pressure. It adapts four different transition structures and four distinct closed pore forms are observed (see Figure 8). In addition to the perfectly diamond-shaped structure, e.g., the 9 × 9 × 9 NC, and a disturbed diamond-shaped structure, e.g., the 5 × 5 × 5 NC, a Z-shaped and a T-shaped closed pore form are observed, respectively shown in Figure 8 for the 6 × 6 × 6 NC and the 8 × 8 × 8 NC.

The four closed pore forms are the result of the four different closing mechanisms. In contrast to the model system DMOF-1 and the DUT-8, the process starts not in one corner but from one side, which induces a layer-by-layer pore closing. The DUT-128 NCs seem to be more flexible, since the direction of pore closing is more spontaneous, e.g., the pore closing of the 5 × 5 × 5 NC ends up in a disturbed diamond-shaped structure, although it shows first a similar layer-by-layer closing like the model system DMOF-1. The second closed pore form is Z-shaped, which means that two interfaces between layers closed along different spatial diagonals can be observed in the closed pore form. The outer layers are closed along the same spatial diagonal, but some of the middle layers are closed along with a different one. The transition structure shows that the pore closing occurs from two sides and spontaneously change the direction, i.e., the pore closes along different spatial diagonals. The third closed pore form is T-shaped, which can be explained by the transition structure indicating a pore closing from three sides along different spatial diagonals. Therefore, there is no possibility to fold the centered pores further to achieve the completely closed form due to the adjacent pores, which are closed along different spatial diagonals. The last closed pore form is the perfectly diamond-shaped structure. The transition structure shows that a symmetrical layer-by-layer closing occurs, i.e., the pore closing is simultaneously in x- and y-direction and propagates from one corner to the opposite one.

Compared to the model system, the DUT-128 NCs are more sensitive to the external pressure mediated by the pressure bath. Therefore, a larger variety of closed pore NC morphologies are seen, and only one mechanism ends up in the lowest energy diamond-shaped closed pore form. In order to arrive in the optimum structure it might be even more important to slow down the linear pressure increase to allow the system to equilibrate. Furthermore, due to the enhanced dispersive interactions between the biphenyl linker backbones in the DUT-128 NCs, the transformation does not initiate exclusively at the corners, but also on entire outer layers of the NC, and pores of middle layers, adjacent to open pore layers, can close spontaneously.

The external pressures needed to initialize the phase transition of the DUT-128 NCs, listed in Table 3, are similar to those needed for the DUT-8 NCs, but lower compared to the model system. They show the same overall trend as observed for DMOF-1 with a substantial size effect for the smaller systems and a convergence of the transition pressure at a size of about 6 × 6 × 6. For the larger systems above 6 × 6 × 6, the external pressure converges around 650 bar, which is the lowest value for all studied MOFs. This convergence is obviously due to a certain number of pores to be closed simultaneously to initiate the phase transformation. Note that also the closing of a corner essentially means that the entire edge along the z-direction of the NC must be transformed. However, further simulations of even larger NCs will be needed to confirm this hypothesis.

**TABLE 3:** The applied external pressures for each system size of DUT-128 NCs at which the phase transition starts. For larger system sizes higher pressures are needed to initialize the closing, roughly converging at a size of 6 × 6 × 6. In general, the necessary external pressures are in the same range for the DUT-8 NCs and lower compared to the model system.

System	Pressure (bar)
3 × 3 × 3	145
4 × 4 × 4	440
5 × 5 × 5	515
6 × 6 × 6	625
7 × 7 × 7	605
8 × 8 × 8	665
9 × 9 × 9	650

It should be noted that at this proof-of-concept stage, all results are based on pressure ramp simulations, carried out once for each investigated system. This gives a good first insight into possible mechanisms of the pore closing process of differently sized MOF NCs with different linkers. In future work, however, several simulations have to be performed to take statistics into account. The non-diamond-shaped closed NCs were observed to be stable structures within the simulation time. However, since the non-reactive force field does not allow for the breaking of coordination bonds, corresponding reaction channels leading to local defects can not be represented in the simulations. A further aspect to be considered is the fact that the formation of non-diamond-shaped morphologies of the closed pore NCs, observed in particular for DUT-8 and DUT-128, could be an artifact due to the relatively quick rise in pressure in the simulations. On the other hand, a close inspection of the volume vs. time curves (see Supplementary Data) reveals that the phase transformation even for the largest NCs is a fast process in the order of 0.1 ns as soon as it is initiated, and a kinetic trapping might be real effect and not due to the simulation conditions. Simulations with a slower pressure increase will have to be performed in order to corroborate the current results.

## 4 Conclusion

A new approach to simulate the breathing phase transition of flexible MOFs beyond periodic boundary conditions is presented. By introducing a pressure bath medium, the transformation from the open to the closed pore form can be investigated in a more realistic manner as compared to previous work, where a distance constraint was used, which implied a reaction coordinate leading to the diamond-shaped structure. Now the pore closing of the NC is triggered by a true hydrostatic pressure, acting equally on the surface without any bias on the closing mechanism. By suppressing attractive interactions between the pressure bath particles and the MOF and a strong vdW interaction within the pressure bath medium it is possible to prevent the medium not to enter the pores of the MOF. With a size of the particles twice of mercury atoms, a compromise between numerical efficiency and smoothness of the cavity surrounding the NC could be achieved.

The pressure bath method was applied to three different MOFs with the underlying topology pcu, which differ in their linker in the x- and y-direction: DMOF-1, DUT-8, and DUT-128. For DMOF-1, four different pore closing mechanisms were observed, which end up in two differently shaped closed pore forms. The DUT-8 NCs show similar closed pore forms as DMOF-1 independent of the linker conformations. The ndc linker affects the external pressure needed to initialize the pore closing, i.e., the pressure has to be lower than for DMOF-1. The expanded linker in DUT-128 leads to more sensitivity concerning external pressure. For the DUT-128 NCs, a larger number of different closed pore forms were observed. The pore closing mechanism differs as well, i.e., the pore closing does not only start in the corners of the NCs but also on the sides. For all systems a size effect in the transition pressure for the small NCs is observed, which converges, however, rather quick at system sizes of about 6 × 6 × 6. It can be concluded that a certain number of pores have to close simultaneously to initiate the transformation.

Future research could include host-guest interactions and their effect on the breathing phase transition. Additionally, the importance of finite size effects needs to be further investigated with even larger NC models, since synthesized MOF powders are still on orders of magnitude larger scales than the here studied systems. An intrinsic limitation of the current methodology is that a pressure mediated reopening of the pores cannot be investigated. For the alternative temperature controlled reopening, the pressure bath properties at higher temperatures have to be explored. Considering the diversity of experimentally determined MOF crystallite shapes, MOF NCs with anisotropic spatial dimensionality will have to be simulated.

The study illustrates that the pressure bath concept allows the exploration of potential energy surfaces for breathing phase transitions of non-periodic MOF NCs. The approach provides a more flexible and realistic alternative to the previously employed mechanical forces, which potentially biases the sampling. Hence, in combination with an extended sampling, and potentially softer pressure ramps, the method could help to clarify the favored pore closing mechanism of flexible MOFs at the molecular scale.

## Data Availability

Simulation input used for this study can be found in the github repository: https://github.com/cmc-rub/supporting_data/tree/master/99-Schaper-PressureBath.
