# Supplementary material for: Molecular Dynamics Simulations of the Breathing Phase Transition of MOF Nanocrystallites II: Explicitly Modeling the Pressure Medium
Source: Front Chem. 2021 Oct 25;9:757680. doi: 10.3389/fchem.2021.757680 (PMC8575409; doi:10.3389/fchem.2021.757680)
Supplement: Supplementary file 1 [file DataSheet1.pdf]

Supplementary Material: Molecular Dynamics Simulations of the Breathing Phase Transition of MOF Nanocrystallites II: Explicitly Modeling the Pressure Medium

1 SUITABLE PARAMETERS FOR THE PRESSURE BATH ATOMS

In order to select suitable parameters for the pressure bath atoms, different combinations of the minimum distance r_0 and the well-depth ϵ parameters were tested. To describe van-der-Waals interactions, the MOF-FF force field uses a dispersion damped Buckingham potential, whose coefficients are calculated out of the minimum distance r_0 and the well-depth ϵ. The here used value for the minimum distance r = 3.0 Å is based on the literature [Bomont, J.-M. and Bretonnet, J.-L. (2006) *J. Chem. Phys.* 124, 054504.], which refers to r = 2.93 Å at the critical point for mercury. For the well-depth parameter ϵ, a screening experiment for different ϵ values from ϵ = 0.1 kcal/mol to ϵ =1.7 kcal/mol in steps of 0.1 kcal/mol was performed using the 4x4x4 system as a test system. For every tested ϵ value, a trajectory of 1 ns without any pressure increasing was calculated in an NpT ensemble after optimization and equilibration of the system in an NVT ensemble.

For ϵ values up to $\epsilon = 0.3$ kcal/mol, a highly increased volume of the simulation box is observed. This could be reasoned in the low interactions between the pressure bath atoms since a well-depth of 0.3 kcal/mol represents a flattened minimum. As a result, the interatomic distance between the mercury atoms increases during the simulation which increases the volume. Additionally, the performance speed of 1.596 ns/day is slower compared to calculations with larger ϵ values. Therefore, these values do not fit for describing the pressure bath which should press on the NC.

For ϵ values higher than ϵ = 1.3 kcal/mol, the NC has a distorted geometry without any external pressure increasing, i.e., the pores are not completely open at the end of the simulation. A longer simulation time may be needed to open all pores entirely, but this would increase computational costs. Therefore, these ϵ values are not suitable for simulations that should describe the structural transition from open to closed pores.

For selected ϵ values in the range of ϵ = 0.4 kcal/mol up to ϵ = 1.2 kcal/mol, a second NpT simulation was performed for 5 ns including a pressure ramp from 0 to 5000 bar, to generate increasing pressure on the system. For each tested ϵ value, it can be observed that the NC folds, i.e., a structural transition from the open pore structure to the closed pore structure occurs. Table S1 shows for the tested ϵ values when the folding process is observed in the trajectory.

The structural transition starts earlier if the ϵ value is increased except for ϵ = 1.2 kcal/mol. To set ϵ = 1.2 kcal/mol seems to be an inefficient choice for the ϵ value of mercury since it needs more steps and higher pressures to simulate the folding process. The efficiency is increased if the ϵ value is increased up to ϵ = 0.9 kcal/mol since fewer steps are necessary to simulate the structural transition of the NC, which shortens the calculations. However, the structural transition starts approximately at the same time for ϵ = 0.8 kcal/mol and ϵ = 0.9 kcal/mol. Due to the faster numerical performance if the well-depth ϵ is set to 0.8 kcal/mol, it was decided to chose ϵ = 0.8 kcal/mol instead of ϵ = 0.9 kcal/mol for further calculations.

Table S1. Time when the structural transition from an open to a closed pore structure in the trajectories calculated with different ϵ values for the pressure bath atoms is observed. For larger ϵ values, the folding process starts earlier. The numerical performance speed (32 processors; 10190 atoms total, thereof 4160 MOF atoms and 6030 pressure bath atoms) is given for the NpT simulation without pressure increasing, and in brackets, for the pressure ramp simulation.

ϵ [kcal/mol]	Time [ns]	Performance speed [ns/day]
0.4	1.23	4.738 [3.829]
0.6	0.95	6.227 [2.832]
0.8	0.83	7.652 [5.018]
0.9	0.70	5.937 [2.631]
1.2	1.04	7.894 [5.896]

Note that the here performed screening experiment does not provide optimized parameters. It was focused on proofing the pressure bath concept meaning we want to show that it is possible to computationally investigate the breathing phase transition under consideration of different pore closing mechanisms. Therefore, it was only searched for a suitable parameter set that works adequately.

2 COMPUTING THE INDIVIDUAL PORE VOLUME OF THE NC

The individual pore volume of the NC is calculated using a convex hull as it is done in the previous work [Keupp and Schmid (2019) *Adv. Theory Simul.* 2, 1900117]. Further information can be found in the corresponding Supporting Information. Starting from the position of the atoms building one paddle-wheel unit, the center of mass (COM) for each paddle-wheel was determined. A convex hull is spanned by eight paddle-wheel COMs that define a pore in the NC, i.e., the convex hull (see Figure S1) encloses the set of COM coordinates. The volume of an individual pore is obtained by calculating the volume of those convex hull.

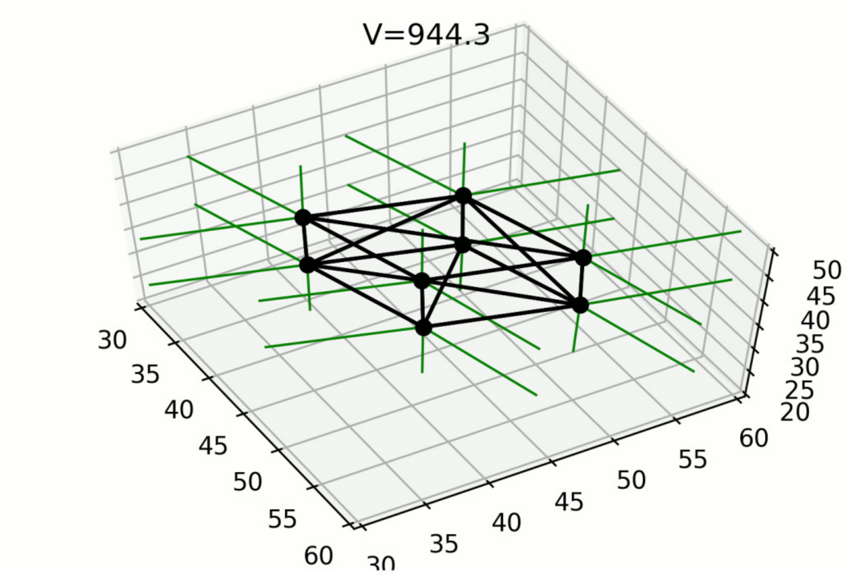

Figure S1. Eight paddle-wheel unit COMs (black dots) define one individual pore. The black lines represent the convex hull resulting in a volume element regarded as the pore volume. The green lines indicate the distance vectors to the paddle-wheel unit COMs. The figure is taken from the Supporting Information of reference [Keupp and Schmid (2019) *Adv. Theory Simul.* 2, 1900117].

3 SIMULATION OF THE BREATHING PHASE TRANSITION OF MOF NANOCRYSTALLITES: VOLUME CHANGE OVER TIME

For the DMOF-1 NCs, the decrease in volume normalized to one pore is plotted against the simulation time in Figure S2. All systems show a sigmoidal curve with a sharp transition from a pore volume of 1170 Å^3 to 610 Å^3. The volume curve of the 9x9x9 system differs due to the interface in which the pore size is larger than in the remaining closed pores, but it ends up at the same closed pore volume. Therefore, the pore closing time correlates with the NC size. This holds up to the 6x6x6 size. For the 6x6x6 NC up to the 9x9x9 NC, the pore closing starts at nearly the same time. Since a pressure ramp simulation was performed, in which an external pressure is increased linearly, the simulation time is directly connected to the applied external pressure, i.e., the external pressure is increasing as time progresses.

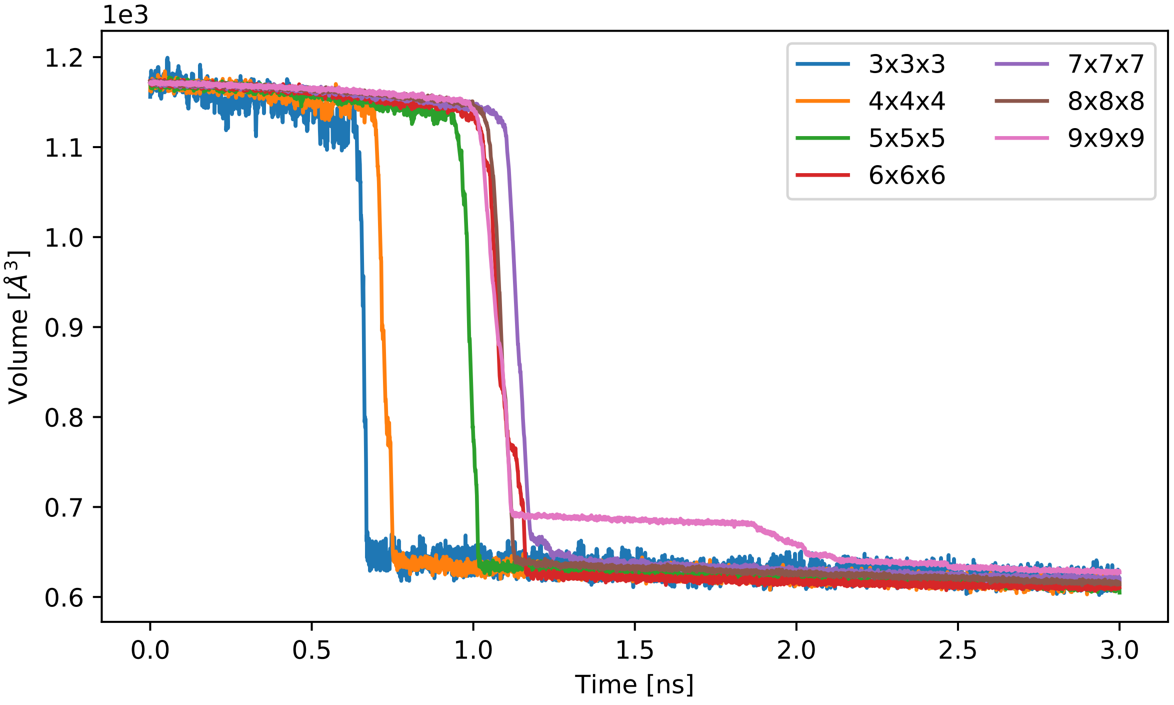

Figure S2. The volume is decreasing during the pressure ramp simulation of the DMOF-1 NCs with the sizes 3x3x3 up to 9x9x9 normalized to the volume of one pore.

For the DUT-8 NCs, the volume change during the simulation is plotted for investigated sizes from the 3x3x3 NC up to the 9x9x9 NC with the respective conformation in Figure S3. The volume decrease during the simulation shows slightly different trends than the model system. For conformation 1 (see Figure S3a), the impact of the system size is the smallest, i.e., the differently sized NCs start the pore closing at nearly the same time, which differs from the model system. For conformation 2 (see Figure S3b) and conformation 3 (see Figure S3c), the phase transitions of the NCs with the sizes 5x5x5 up to 9x9x9 occurs nearly simultaneously, i.e., in DUT-8 NCs, the system size is less critical for the initialization point than in the model system. For conformation 4 (see Figure S3d), the phase transition of the 5x5x5 and the 6x6x6 NC occurs nearly simultaneously, whereas, for the 7x7x7 NC up to the 9x9x9, the phase transitions require higher pressure to be initialized. However, independent of the conformation, most DUT-8 NCs start their phase transition between 0.5 ns and 1.0 ns, depending on the system size. An exception is the 3x3x3 NC with the conformation 3 (see Figure S3c), which starts before 0.5 ns.

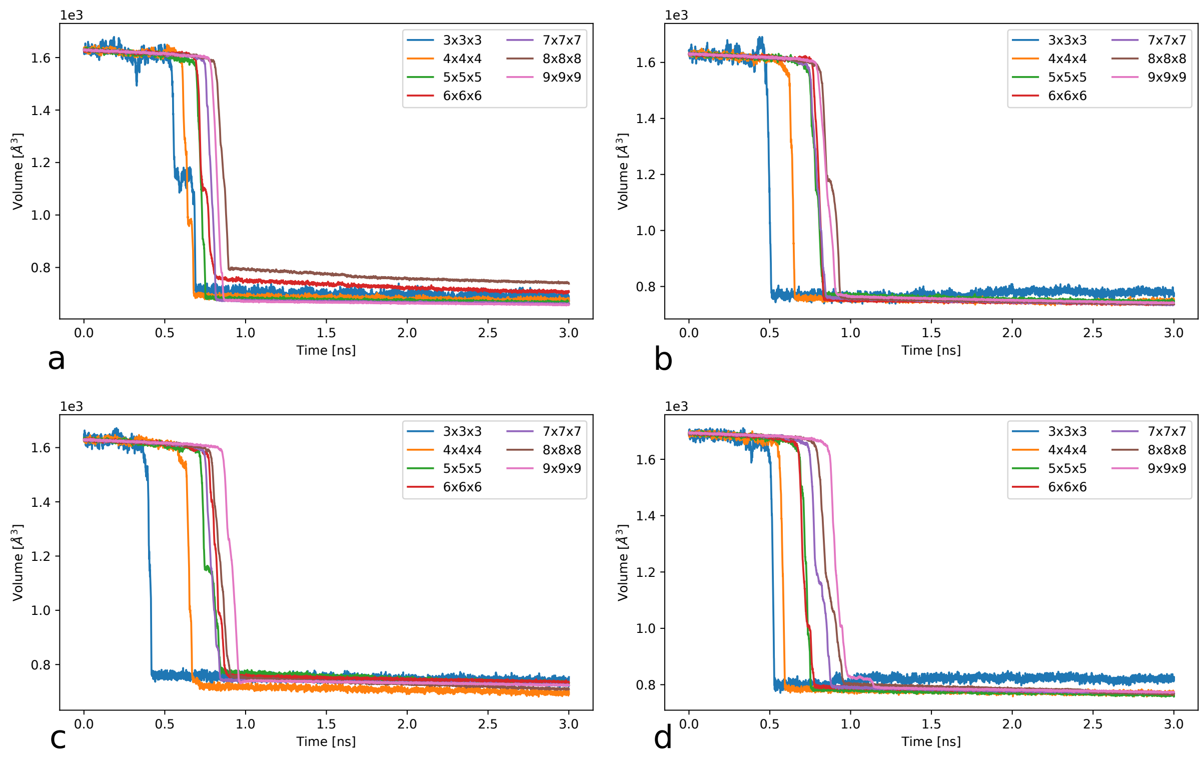

Figure S3. The volume is decreasing during the pressure ramp simulation of the four investigated conformations of DUT-8 NCs with the sizes 3x3x3 up to 9x9x9 normalized to the volume of one pore. Independent of the conformation, most phase transitions start between 0.5 ns and 1.0 ns.

In Figure S4, the total volume of the NCs of the DUT-128 NCs is plotted against the simulation for each system and normalized to the size of one pore. All phase transitions, in which the pore volume decreases from 2250 Å^3 to 700 Å^3, occur between 0.5 ns and 1 ns except for those of the 3x3x3 NC, which is similar to the 3x3x3 DUT-8 NC with conformation 3. The curves overlap for the 6x6x6 NC up to the 9x9x9 NC, i.e., they close nearly simultaneously. For the 4x4x4 NC (orange), the 5x5x5 NC (green), and the 7x7x7 NC (purple), the curves show small plateaus, which means that the pores do not close as coherently as in the model system. This could be caused by the pore closing along different spatial diagonals. Higher pressures are needed to close the interfacial pores completely, which are provided later by the linear pressure increasing during the simulation.

4 ADDITIONAL PORE SIZE ANALYSES FOR DUT-8 AND DUT-128

The DUT-8 and the DUT-128 NC show additional pore closing mechanisms compared to the model system DMOF-1. To sketch them in detail, pore size analyses were performed equivalent to the procedure performed for the model system. Due to the ndc linker in DUT-8 and the bpdc linker in DUT-128, the individual pore size is larger than for DMOF-1 constructed with the bdc linker.

For DUT-8, blue-colored pores represent closed pores with a volume of approximately 1800 Å^3, whereas red-colored pores represent the open pores with a volume of approximately 610 Å^3. The first additional pathway shown in Figure S5a for the 7x7x7 NC with the conformation 1 starts at one corner and propagates first in one direction and then in the other direction. At t = 0.790 ns, the top layer is entirely closed, which induces that at t = 0.798 ns, a middle layer adjacent to open pore layers, close its pores. Since all pores are closed along the same spatial diagonal, the two remaining layers close along those spatial diagonal to

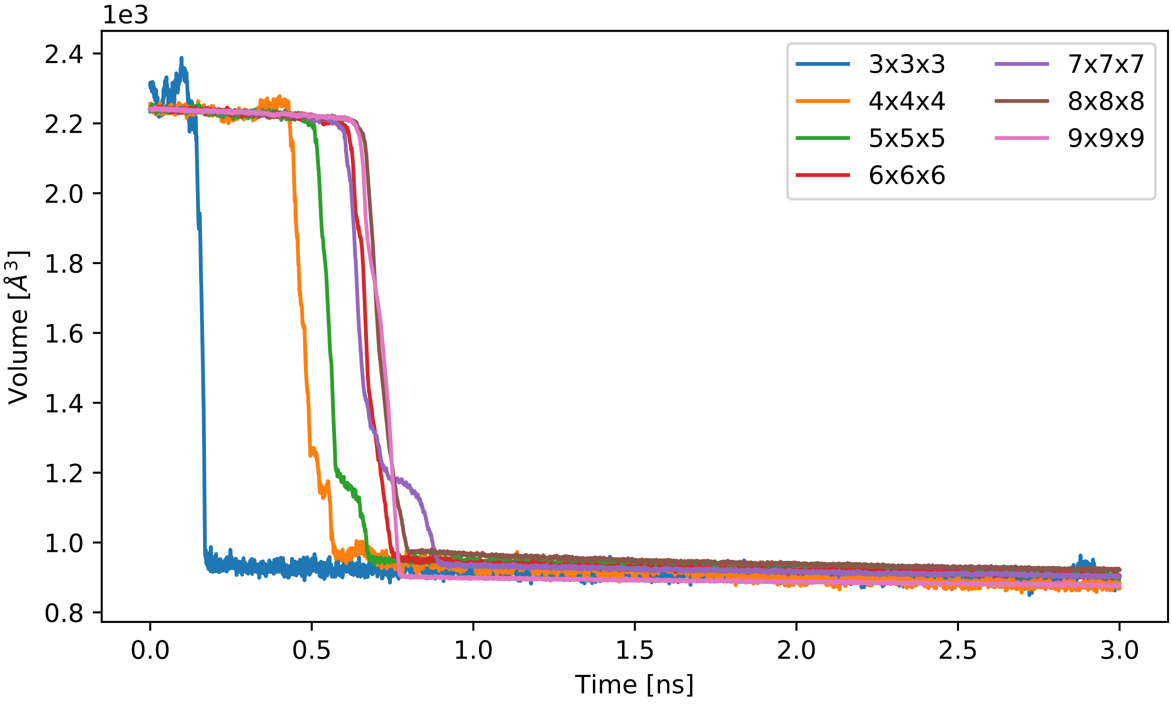

Figure S4. The volume is decreasing during the pressure ramp simulation of the DUT-128 NCs with the sizes 3x3x3 up to 9x9x9 normalized to the volume of one pore. Up to the 6x6x6 NC, the phase transition starts later if the system size is increased. For the 6x6x6 NC up to the 9x9x9 NC, the pore closing is initialized nearly simultaneously.

end up in the diamond-shaped structure. The second additional pathway is shown in Figure S5b for the 7x7x7 NC with the conformation 4. From t = 0.770 ns up to t = 0.850 ns, the pore closing occurs from two opposite corners along different spatial diagonals. This leads to a partial diamond-shaped transition structure. The remaining pores close either along with the one or the other spatial diagonal, which is why the structure is bent on the top and the right side up to t = 0.860 ns. If the pressure is further increased, all pores are completely closed, but the structure keeps the disturbed diamond-shaped structure.

For DUT-128, blue-colored pores represent closed pores with a volume of approximately 2250 Å^3, whereas red-colored pores represent the open pores with a volume of approximately 700 Å^3. The first pathway resulting in the disturbed diamond-shaped structure is shown in Figure S6a for the 5x5x5 NC. The pore closing does not start at the corner of the NC as it is always observed for the model system but at one site. At t = 0.525 ns, one pore on the left side of the NC is completely closed. Up to t = 0.575 ns, the NC closes layer-by-layer induced by the adjacent closed pores. From t = 0.600 ns up to t = 0.700 ns, there are two competing effects affecting the pore closing of the still open outer layer. In addition to the adjacent closed pores, the pressure bath medium mediates pressure to the outer layer. As a result, the outer layer on the right closes along another spatial diagonal ending in the disturbed diamond-shaped structure. The second mechanism has a spontaneous character, as it is shown in Figure S6b for the 6x6x6 NC. After the pore closing of one side, it continues in one of the middle layers as it can be seen for t = 0.675 ns up to t = 0.712 ns. This middle layer closes along a different spatial diagonal. Therefore, the direction of the pore closing changes spontaneously. In addition, the outer layer opposite to the first closed layer starts its pore closing simultaneously, i.e., the pressure bath medium induces the pore closing of this outer layer. Thus, the Z-shaped closed pore form results from two interfaces between layers closed along different spatial

diagonals. The outer layers are closed along the same spatial diagonal, but some of the middle layers are closed along a different one. The third mechanism, shown in Figure S6c for the 8x8x8 NC, starts at one side similar to the formerly described ones. This induces the pore closing along another spatial diagonal of a nearly orthogonal middle layer from t = 0.685 ns up to t = 0.700 ns. At t = 0.725 ns, the pores of the opposite corner start to close along a third spatial diagonal. The pore closing continues from three directions to the centered pores resulting in the T-shaped closed pore form. At t = 0.800 ns, the centered pores have a larger pore volume than the remaining closed pores, which does not become smaller if the pressure is further increased. This can be explained by the geometry of the closed pore form. There is no possibility of folding these pores further to get the completely closed form due to the adjacent pores closed along different spatial diagonals. The fourth observed pathway, shown in Figure S6d, ends up in the perfectly diamond-shaped structure. Starting in one corner, the pore closing continues simultaneously in the x- and y-direction up to t = 0.725 ns. Then, the pore closing is fast in one direction. Despite this, all pores close along the same spatial diagonal. No pore closing is observed on other sides of the NC, which can be interpreted as a smaller impact of the pressure bath for this pore closing mechanism.

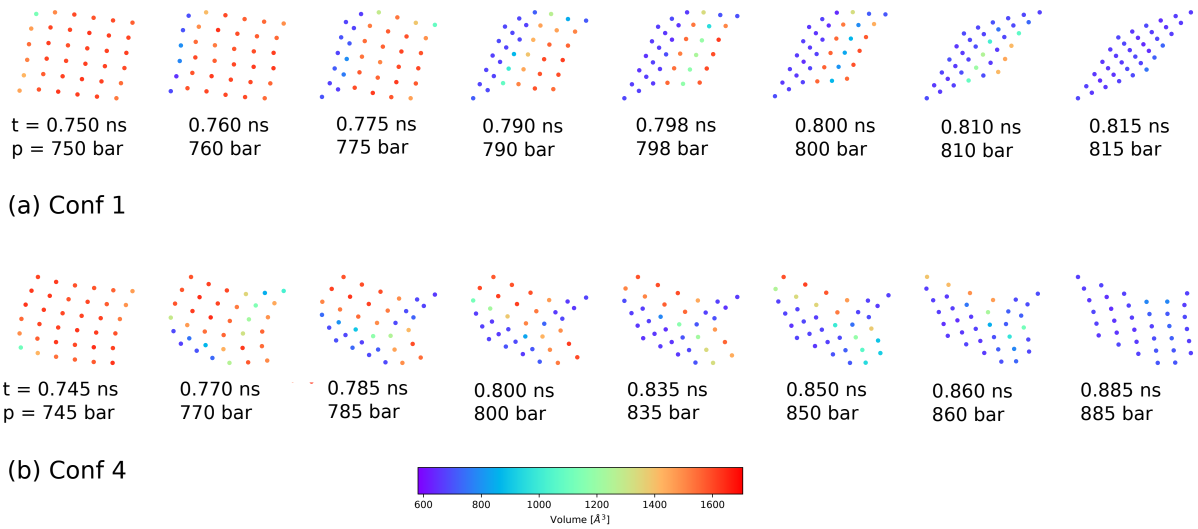

Figure S5. Pore size analysis of the 7x7x7 DUT-8 NC with the conformation 1 and 4. (a) The pore closing starts in the upper left corner and propagates in an asymmetrical layer-by-layer closing through the NC. Remarkably, the top layer closes fast, which induces a pore closing of a middle layer adjacent to open pore layers. Since all pores are closed along the same spatial diagonal, the closed pore form is the diamond-shaped structure. (b) The pore closing co-occurs from two corners along different spatial diagonals. This leads to a disturbed diamond-shaped structure.

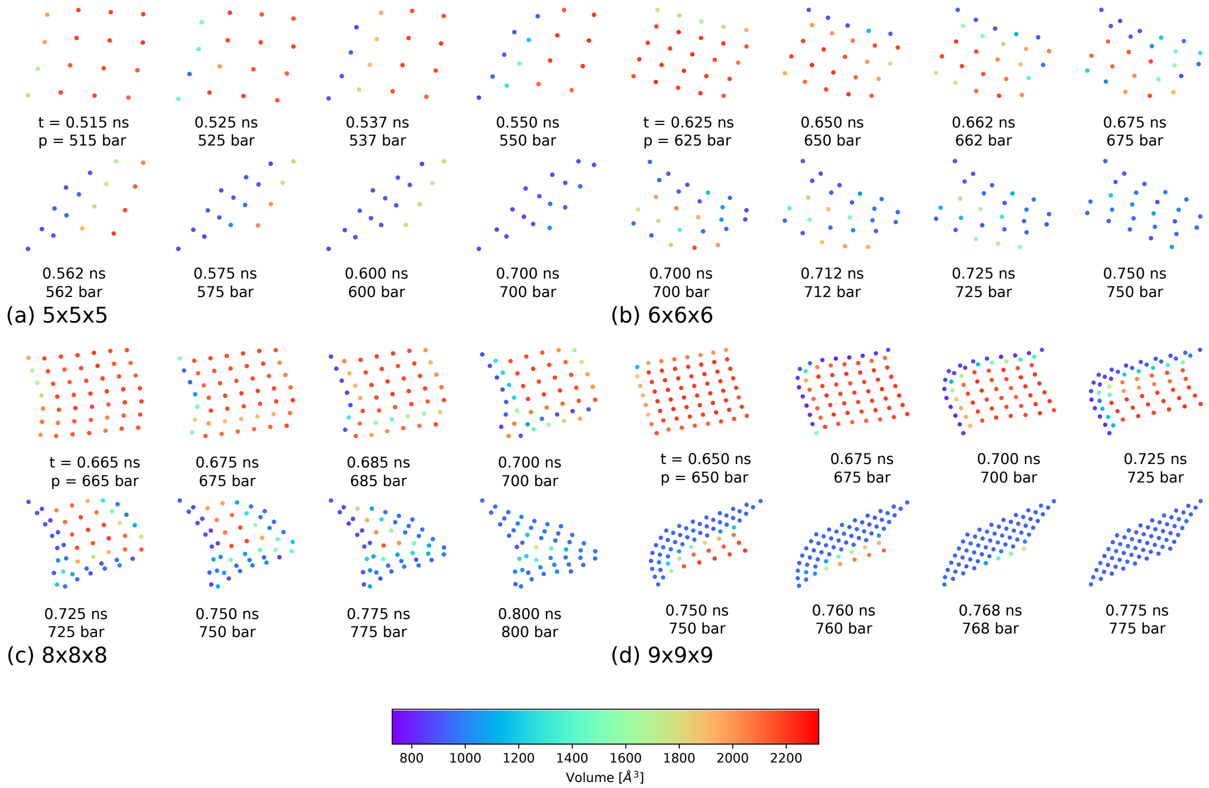

Figure S6. Pore size analysis of the differently sized DUT-128 NC for different snapshots of the phase transition from open to closed pore structure averaged along the z-axis and viewed on the xy-plane. (a) For the 5x5x5 NC, the center of the pores is colored according to the respective size. The phase transition starts at one layer and induces a layer-by-layer closing. The last layer closes along a different spatial diagonal so that the closed pore form is a disturbed diamond-shaped structure. (b) For the 6x6x6 NC, the center of the pores is colored according to the respective size. In this pore closing mechanism, the layers inside the NC are closed, although their adjacent layers are still open. The Z-shaped closed pore structure results from adjacent layers closed along different spatial diagonals if the pressure increases. (c) For the 8x8x8 NC, the center of the pores is colored according to the respective size. The pore closing travels from three sides to the centered pores. Since the pores closing along different spatial diagonals, the centered pores cannot be completely closed. (d) For the 9x9x9 NC, the center of the pores is colored according to the respective size. A symmetrical pore closing mechanism is observed, i.e., it starts at one corner and travels along the diagonal of the NC to the opposite corner to end up in the diamond-structure.
